# Phospholipid supplementation inhibits male and female odor discrimination in mice

**DOI:** 10.3389/fnbeh.2024.1397284

**Published:** 2024-07-25

**Authors:** Maryana Morozova, Jelizaveta Andrejeva, Olga Snytnikova, Lidiya Boldyreva, Yuri Tsentalovich, Elena Kozhevnikova

**Affiliations:** ^1^Scientific Research Institute of Neurosciences and Medicine (SRINM), Novosibirsk, Russia; ^2^Institute of Molecular and Cellular Biology SB RAS, Novosibirsk, Russia; ^3^International Tomography Center SB RAS, Novosibirsk, Russia; ^4^Laboratory of Bioengineering, Novosibirsk State Agrarian University, Novosibirsk, Russia

**Keywords:** phospholipids, diet, metabolism, odor discrimination, social behavior

## Abstract

Dietary phospholipids (PLs) are promising supplements that are commonly found as natural food ingredients and emulsifier additives. The present study aimed to evaluate the effect of major PLs found in food supplements on social behavior in mice. In this study, the effect of short-term high dietary PL content was studied in terms of social odor discrimination and social interactions with male and female intruders in male mice. We used odor discrimination and habituation tests to demonstrate that PL-fed male mice tend to lose preference toward female odor and fail to discriminate against socially significant scents. At the same time, test animals recognize non-social odors. We also found that PL affected the social behavior of the test males, who tend to behave indiscriminately toward male and female intruders during direct contact. Brain metabolomic profiling revealed no major changes in the intermediary metabolism or neurotransmitter biosynthesis. At the same time, intranasal PL application resembled the effects of dietary supplementation. These data suggest that certain PL might suppress pheromone perception in the olfactory system and affect the sense of socially important odor cues.

## 1 Introduction

Phospholipids (PL) constitute a category of complex lipids characterized by the presence of a phosphate group joined to a pair of fatty acid moieties via a glycerol linkage (Harayama and Riezman, 2018; Boldyreva et al., 2021). Lipid molecules tend to naturally form bilayers that contribute to the structure of cellular organelles and the plasma membrane (Drin, 2014). Additionally, phospholipid (PL) molecules and their metabolic products play a crucial role in various signaling pathways, functioning as secondary messengers, and regulating essential cellular processes ranging from cell growth to detecting smells (Klasen et al., 2010; Musille et al., 2013; Crowder et al., 2017). A broad consensus supports the beneficial impact of dietary PL supplementation on human health and the management of various diseases, including neurological disorders (Kullenberg et al., 2012).

Due to the proven beneficial impact of PL on human health and their various physiological functions, a wide variety of PL-enriched food supplements are available in the market (Kullenberg et al., 2012; Kutzner et al., 2017; Bernhard et al., 2020). These PL-based additives can be purchased without a prescription and are generally considered safe and advantageous for human well-being (Jorissen et al., 2002; Glade and Smith, 2015). Most of these dietary supplements contain phosphatidylcholine (PC) as their primary component, at concentrations ranging from 30 to 100%, with natural soy lecithin being among the most widely used one. Furthermore, soy lecithin is commonly utilized as an emulsifying agent in everyday food production (Gholmie et al., 2020; Nemati et al., 2021). As a result, on average, regular diets include substantial quantities of PL from both synthetic and natural sources.

We have previously shown that long-term supplementation of regular laboratory mouse chow with major PL available as dietary supplements inhibits social odor discrimination and impairs social interactions in male mice (Boldyreva et al., 2024). This study aims to assess the effect of short-term PL feeding on odor cue perception and male–male and male–female communication in mice.

## 2 Materials and methods

### 2.1 Animals

The study was conducted at the Scientific Research Institute of Neurosciences and Medicine (SRINM) and adhered to the regulatory framework of Russian law, including Good Laboratory Practice as outlined in Directive # 267 from 19 June 2003 by the Ministry of Health of the Russian Federation, the institutional ethical committee’s guidelines, and the European Convention for the protection of vertebrate animals. Ethical approval was granted by the SRINM’s Ethical Committee, denoted by Protocol #3, on 19 May 2022. All animals were maintained with the Specific Pathogen Free (SPF) status, verified through quarterly tests in accordance with the recommendations by the Federation of European Laboratory Animal Science Associations (FELASA) (Borisova et al., 2020). The research utilized the C57BL/6JNskrc strain, our in-house derivative of the C57BL/6 J colony.

The study involved adult male mice, aged between 8 and 12 weeks, housed in individually ventilated cages (Optimice, Animal Care Systems, Düsseldorf, Germany) containing birch sawdust for bedding and paper cups for enrichment. The photoperiod in the facility followed a 12-h light/dark cycle [12:00 (OFF): 00:00 (ON)]. The test animals had continuous access to food (BioPro, Novosibirsk, Russia) and water. For social tests, 10 adult sexually experienced male and 10 estrus female BALB/c mice of the same age served as intruders and as a source of soiled bedding. BALB/c mice were selected due to their easily recognizable fur color compared to the test C57BL/6 mice.

For short-term PL treatment, standard chow (BioPro, Russia) was mixed with PL dietary supplement capsules in a composition of 840 mg of PC (Solgar, Leonia, NJ, United States) and 100 mg each of phosphatidic acid (PA) and phosphatidylserine (PS) (4 + NUTRITION, Padova, Italy) for every 30 g of the enriched feed. An average mouse weighing 25 g consumes approximately 5 g of chow daily, which results in 5600 mg of PC, 680 mg of PS, and 680 mg of PA per kg per day. According to the mouse chow manufacturer (BioPro, Novosibirsk), the regular laboratory mouse diet contains 6% fat. Fat is mainly derived from full-fat soy, sunflower oil, sea fish fat, corn gluten, and meat and bone meal. Above 50% of the total fat originates from soy, and approximately 15% is sunflower oil. The exact PC, PS, and PA content was not provided by the manufacturer but was approximated from published data given the major fat constituents of the chow (Mad'yarov et al., 1994; Thavarith Chunhieng et al., 2008; Cohn et al., 2010; Cui and Decker, 2016) as follows: 375 mg of PC, 43 mg of PS, and 31 mg of PA per kg daily.

A 2-week PL supplementation started by feeding 8–10-week-old male mice with the described PL-enriched diet and continued throughout the behavioral tests. At the end of the experiment, the mice were humanely euthanized via cervical dislocation, and their brains were harvested for metabolic analysis. Metabolites were extracted and analyzed using nuclear magnetic resonance (NMR) techniques. Food intake was not assessed during the experiment, but there was no difference in average weight between the animal groups at the end of the experiment.

For intranasal PL treatment, 840 mg of PC (Solgar, Leonia, NJ, United States), as well as 100 mg each of phosphatidic acid (PA) and phosphatidylserine (PS) (4 + NUTRITION, Padova, Italy), were vigorously resuspended in 9 mL of PBS, and 15 μL of the suspension was applied intranasally (approximately 7.5 μL per nostril) to 10–12-week old sexually experienced C57BL/six male mice 30 min prior to the test. A control group of 10 sexually experienced C57BL/six male mice of the same age was subjected to intranasal application of 15 μL of PBS supplemented with glycerol at a concentration corresponding to that in PL solution. PL was administered to both nostrils. The dose was calculated to recapitulate the proportion of dietary PL to the animal’s weight (assuming the weight of the olfactory organs exposed to intranasal PL is approximately 1/100 of the total weight). The final doses of the PL were estimated as follows: 5,600 mg of PC, 667 mg of PS, and 667 mg of PA per kg of the affected tissue per day. The 30-min interval was chosen to ensure that we observe the primary effects of the PLs but not the secondary transcriptional events.

### 2.2 Spectroscopy

The metabolome of the brain samples (*n =* 6 per group) was studied at the Collective Use Center “Mass spectrometric investigations” of the Siberian Branch of the Russian Academy of Sciences (SB RAS) in Novosibirsk, Russia, using high-resolution 1H NMR spectroscopy. One-half of each brain dissected in the sagittal plane was used as a sample. Metabolite extraction from the brain followed a previously validated brief protocol for quantitative NMR metabolomic analysis (Morozova et al., 2022). In summary, brain tissues were placed in 1.5 mL Eppendorf tubes, flash frozen in liquid nitrogen, weighed, and then homogenized in a cold (−20°C) mixture of water, methanol, and chloroform at a 1:2:2 volume ratio, using 1,600 μL per 150 mg of tissue. After homogenization, the mixtures were vortexed, cooled with ice, and then incubated at −20°C, followed by centrifugation to separate proteins. The aqueous layer was collected, dried by a vacuum concentrator, and then dissolved in 600 μL of deuterated water containing sodium 4,4-dimethyl-4-silapentane-1-sulfonate (DSS) as a standard and a deuterated phosphate buffer to maintain a neutral pH. The samples were analyzed on a Bruker BioSpin AVANCE III HD 700 MHz NMR spectrometer with a cryomagnet. The 1H NMR spectra were recorded with multiple scans at a consistent temperature of 25°C using a 90° detection pulse, pre-saturation for the water signal, and adequate relaxation time between scans. Manual spectrum adjustments such as phasing and baseline correction were implemented, followed by signal integration via MestReNova V.12 (Mestrelab Research S.L.) software. Metabolite identification was cross-referenced against the human metabolome database (accessed on 12 January 2024)^1^ and previous metabolomics studies, with additional validation by introducing standard chemical compounds as appropriate. Quantification was based on the integration of peak area relative to the DSS standard.

### 2.3 Behavioral tests

Behavioral tests were performed in sexually experienced male mice, which were single-housed in open cages (with a dimension of 318 × 202 × 135 mm, #CP-3, 3 W, Russia) 7 days prior to the first behavioral experiment. The interval between tests was 2–3 days. Short-term PL treatment was performed in two separate experiments, with 10 animals per group in each experiment. The results of the two were combined. In the first experiment, only the odor preference and the two-intruders tests were conducted. In the second experiment, the animals were tested in all behavioral paradigms described, followed by testosterone and brain metabolomic analyses. The total number of animals was 18–20 in the two-intruders test, 17–19 in the odor preference test, and 10 animals per group in all other tests. Some animals from the first experiment were excluded from the analysis based on the following criteria: total odor/intruder investigation times were <10 s and showing highly aggressive behavior toward a female intruder, which resulted in wounding (one control male). The behavioral tests were performed in the following order: the open field test, the odor preference test, the odor discrimination and habituation tests, and the two-intruders test.

#### 2.3.1 Open field test

The open-field test is utilized to evaluate both the locomotor and exploratory behaviors of the test animals and also to measure the overall anxiety levels. For this experiment, a mouse was introduced into the center of a clear-walled, opaque-bottomed 40 × 40 cm square arena under white illumination. The inner 20 × 20 cm region was designated as the central zone. An overhead video camera captured the mouse’s locomotion, whereas a separate camera positioned laterally tracked its vertical movements for 6 min. Analytical parameters included the accumulated distance traveled, time spent in the central area, the frequency of rearing events, and the number of center zone entries, all of which were assessed with the aid of Ethovision XT10 software. The test sample size was 10 animals per group.

#### 2.3.2 Odor preference test

The odor preference assay enables the assessment of an animal’s attraction/aversion to different scents without direct contact with the source of the odor. Bedding samples from both female and male BALB/c mice were placed into the test animal’s home cage in two tea infusers (Ikea, art. #469.568.00) for 5 min. The BALB/c mice were housed in their respective cages for 1 week prior to the experiment to ensure that the bedding was sufficiently soiled. An active investigation involving characteristic movements of the nose and whiskers was recorded as “sniffing” behavior and was evaluated manually by two observers. In the short-term PL treatment experiments, the observers were blinded to the group allocation. In the intranasal treatment experiment, no blinding was used. The data were calculated as the ratio of the time spent sniffing each sample to the total time spent sniffing and are presented in percentages. The test sample size was 17–19 animals per group.

#### 2.3.3 The two-intruders tests

This experiment aimed at exploring aspects of social behavior in mice, such as social interaction, aggression, and mating. Test males were isolated in individual cages for a minimum of 3 days before the test. Male and female intruder BALB/c mice had prior sexual experience. During the evaluations, both male and female intruders were introduced into the resident animal’s cage simultaneously for a duration of 15 min. Physical encounters like attacks and mating were counted manually in real-time by two observers, with the results being averaged. Indicators of social interest, such as the frequency and time of sniffing, grooming, following, and chasing, were calculated via a video recording by the same observers and subsequently averaged. The time of sniffing (nasal and genital), grooming, chasing, and following were calculated together and are further described as the time of contact and expressed as a percentage of the total contact time with a male and a female intruder. The test sample size was 18–20 animals per group.

#### 2.3.4 Odor discrimination, olfactory habituation, and olfactory memory

For these tests, the home cage lid was substituted with a plastic cover with a hole for accommodating pipette tips that were used to place cotton swabs soaked in odor samples. For olfactory habituation and discrimination of non-social odors, 0.01% acetophenone (#42163, Sigma-Aldrich, Missouri, United States) and 0.01% lemon flavor (Lemon, Golden Garden, Spain) were used. First, acetophenone was presented five consecutive times for 3 min each at a 1-min interval. Then, the lemon flavor was presented for 3 min. After 60 min, acetophenone was presented for another 3 min. For the female odor discrimination test, diestrus female urine was presented three consecutive times for 2 min at a 1-min interval. This session was followed by the estrus female urine, which was presented in the same manner as the diestrus urine sample. The test sample size was 10 animals per group.

#### 2.3.5 Testosterone measurement

Blood samples (*n =* 10 in each group) were collected using an orbital sinus puncture and centrifuged at 3,000 rpm for 15 mi at room temperature. Then, 20 μL of blood sera were used to measure testosterone by ELISA using the commercial kit according to the manufacturer’s protocol (X-3972, Vector-Best, Novosibirsk, Russia).

#### 2.3.6 Statistical analyses

The data are presented as boxplots with overlaying data points or bar graphs for percentages. The Kolmogorov–Smirnov test was employed to test for normal distribution. Data that followed a normal distribution (including planned comparisons) underwent the Student’s *t*-test for independent or dependent samples, followed by a Holm–Bonferroni adjustment for multiple comparisons when applicable. For female odor discrimination and odor habituation and discrimination tests, an ANOVA with subsequent planned comparisons was used. Non-normally distributed data were examined using the Mann–Whitney *U*-test. The percentage of animals that attacked or mated in the two-intruders test was analyzed using the Fisher exact test. Metabolomic data were analyzed using principal component analysis (PCA) of data that was mean-centered, divided by the standard deviation, median-normalized, and corrected for multiple comparisons using the false discovery rate (FDR). Metabolomic data analyses, including PCA and volcano plots, were performed with MetaboAnalyst 6.0 (accessed on 16 January 2024).^2^ A *p* < 0.05 was considered indicative of statistical significance.

## 3 Results

In many prominent PLs, PC is the most prevalently used component in dietary supplements due to its natural abundance in various foods and in the plasma membranes of mammals (Vance, 2015; van der Veen et al., 2017). This study utilized PC as the primary component in the PL supplementation regimen, constituting approximately 80% of the total PL administered. Additionally, PA—the fundamental precursor to all PLs—was included at a proportion of approximately 10%. PS, another PL component commonly found in commercial food supplements, constituted the remaining 10% of the PL mixture incorporated into the animal feed (Jorissen et al., 2002; Hirayama et al., 2014). Therefore, the experimental mouse groups were fed a modified diet that contained 34.6 g/kg of this PL mixture in their standard chow, with a PL ratio of PC:PA:PS of 8.4:1:1.

### 3.1 Short-term PL treatment impairs social odor discrimination and reduces female preference in male mice

**Figure 1:**
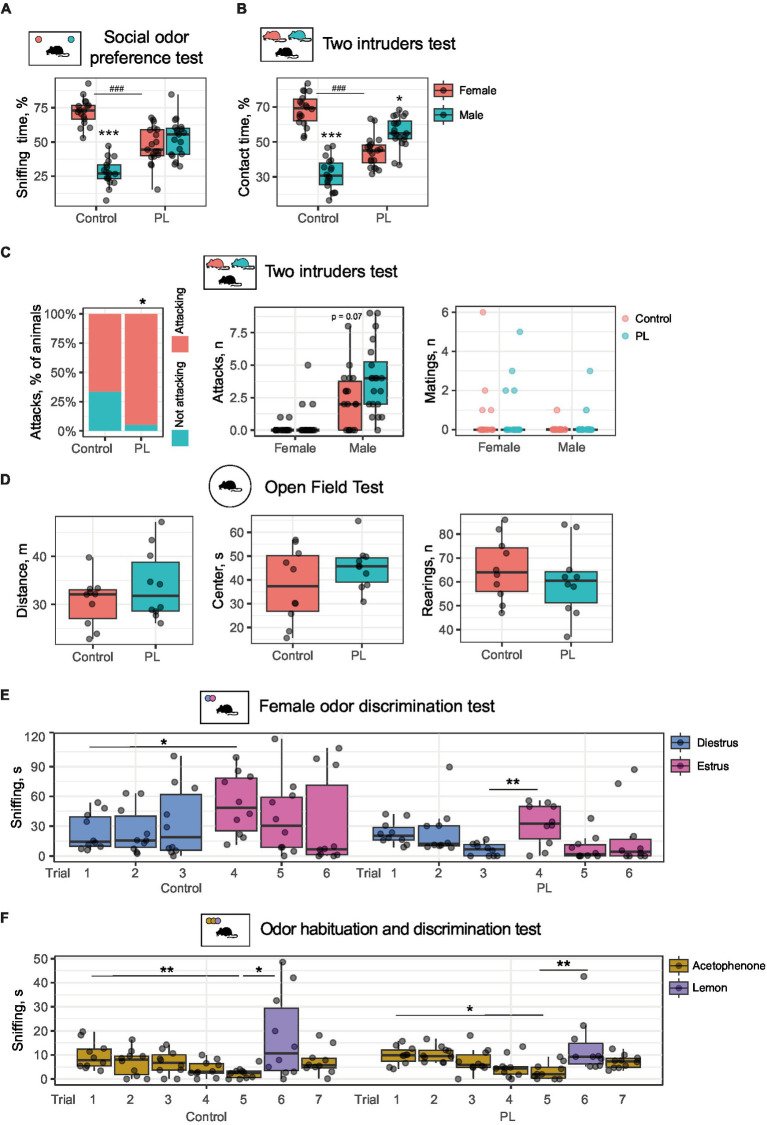
Short-term PL treatment impairs social odor discrimination and reduces female preference in male mice. **(A)** Odor preference test. Control mice prefer sniffing female bedding samples, whereas PL-treated mice demonstrate no preference. ^*^Male vs. female, ^#^control vs. PL. **(B)** The two-intruders test. Control mice prefer a female intruder, whereas PL-treated males interact more with a male intruder. ^*^Male vs. female, ^#^control vs. PL. **(C)** The two-intruders test. The number of attacks and the percentage of animals with aggressive behaviors toward male intruders. **(D)** Open field test: PL treatment did not affect locomotor activity. **(E)** Female odor discrimination test. PL treatment affected interest in estrus female urine. **(F)** Odor habituation and discrimination. Both control and PL-treated males habituate to the odor of acetophenone and discriminate between it and lemon odor. ^*^p < 0.05, ^**^p < 0.01, ^***,###^*p* < 0.001.

Our previous experiments demonstrated that prenatal and the following long-term PL dietary supplementation impaired socially relevant odor discrimination in mice (Boldyreva et al., 2024). In this study, we tested whether short-term PL treatment is sufficient to induce similar changes in olfactory sensing and processing. After the 1-week PL feeding period, male mice were first subjected to the social odor preference test, where we used soiled bedding from male and female cages as the odor source. Control male mice prefer to investigate female bedding samples, which results in a longer time of sniffing [*t*(16) = 9.38, *p*(adjusted) < 0.001, using Student’s *t*-test for dependent samples]. On the contrary, PL-treated mice did not prefer female odor. Test males also spent less time exploring female samples than the control males [*t*(34) = 6.33, *p*(adjusted) < 0.001, using Student’s *t*-test for independent samples] (Figure 1A). This finding suggested that even short-term PL treatment has a strong effect on odor recognition in male mice.

Subsequently, we tested if the PL-supplemented diet also affected direct interaction with male and female intruders. For this purpose, we used the two-intruders test, in which a resident animal is in direct contact with a male and a female intruder simultaneously. Thus, the time of contact with either counterpart can be compared directly. As expected, the control animals that consumed regular chow preferred to follow and investigate a female intruder [*t*(17) = 8.58, *p*(adjusted) < 0.001]. In contrast, the PL-treated animals spent substantially more time in contact with a male intruder than with a female one [*t*(19) = 2.94, *p*(adjusted) = 0.01, using Student’s *t*-test for dependent samples]. In addition, PL-treated males showed less interest in a female intruder than the control males [*t*(36) = 8.33, *p*(adjusted) < 0.001, using Student’s *t*-test for independent samples] (Figure 1B). An increase in aggressive behavior toward male intruders was also notable upon PL supplementation (Figure 1C). The percentage of animals that attacked males was higher in the PL group (*p* < 0.05, using the Fisher exact test). The number of attacks per animal showed an increasing trend. However, no increased aggression toward females was found, suggesting that some discrimination between males and females remains in PL-treated mice. Similarly, PL supplementation had no effect on mating behavior toward either male or female intruders.

These data agree with our previous findings that a PL-supplemented diet impairs socially relevant odor perception and social behavior (Boldyreva et al., 2024). An open field test revealed no differences in activity upon PL treatment (Figure 1D), so the described above social impairments cannot be attributed to general locomotor dysfunction.

### 3.2 Short-term PL treatment affects interest in social odors but not in other scents

We then questioned whether PL-treated animals could discriminate between other socially significant odors or non-social scents. First, we tested urine samples from either diestrus or estrus females for discrimination by the control and PL-treated males. During the test, a male was exposed to a diestrus urine sample in three serial trials, followed by an equal number of presentations with the estrus urine sample. There was an effect of interaction between the trial and PL supplementation on the sniffing time [*F*(1, 108) = 32.04, *p* = 0.04, using a three-way repeated measures ANOVA]. We found that the control animals distinguished between estrus and diestrus samples at first presentation and sniffed more of the estrus female’s urine odor [*t*(9) = 3.04, *p*(adjusted) = 0.03, using Student’s *t*-test for dependent samples]. We also expected a stronger rate of habituation in the estrus sample, but we observed only a slight decreasing trend in sniffing time with repeated presentations. The lack of habituation is probably due to the low number of trials for the control animals. PL-fed mice showed no increased interest in estrus urine samples at first presentation (Figure 1E). At the same time, there was an increase in interest in the new odor between Trials 3 and 4 [*t*(9) = 3.81, *p*(adjusted) = 0.02, using Student’s *t*-test for dependent samples]. This result supports our previous finding in the two-intruders test that PL-treated males retain some capability to discriminate between social scents. It is important to note that we found no statistical interaction between PL and urine type. Thus, PL is likely to generally affect interest in social odors (Figure 1E).

At the same time, when subjected to non-social odors such as acetophenone and lemon scent, both the control and the PLs-fed animals demonstrated successful habituation and discrimination of these odors (Figure 1F). Acetophenone was provided to both groups in five trials, followed by a single lemon scent trial and a final acetophenone trial. There was an interaction effect between the odor and the trial factors on sniffing time [*F*(5, 126) = 10.11, *p* = 0.02, using a three-way repeated measures ANOVA]. Serial presentations of acetophenone were accompanied by a decrease in interest indicative of habituation in both groups [Control, Trial 1 vs. Trial 5: *t*(9) = 4.72, *p*(adjusted) = 0.003; PLs, Trial 1 vs. Trial 5: *t*(9) = 2.7, *p*(adjusted) = 0.03]. Subsequent lemon scent increased sniffing time, suggesting efficient odor discrimination in both groups [Control, Trial 5 vs. Trial 6: *t*(9) = 3.01, *p*(adjusted) = 0.03; PLs, Trial 5 vs. Trial 6: t(9) = 5.52, *p*(adjusted) = 0.001, using Student’s *t*-test for dependent samples]. However, there was only a decreasing trend in both groups following another acetophenone sample. These results demonstrate that there is no difference between the groups in habituation and discrimination of non-social scents. Thus, PL only affects the perception and processing of social odors but no other odors.

### 3.3 Short-term PL treatment does not affect brain intermediary metabolism

**Figure 2:**
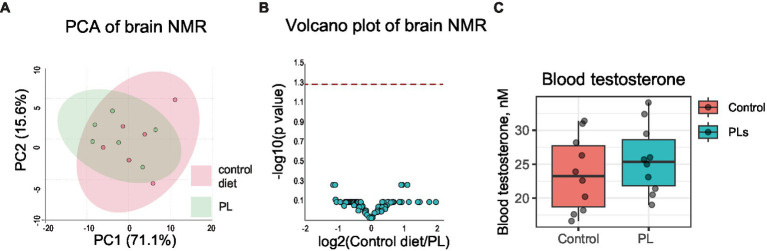
Short-term PL treatment does not affect brain intermediary metabolism or blood testosterone. Untargeted brain NMR metabolomic data was analyzed using MetaboAnalyst6.0 (accessed on 16 January 2024). **(A)** Principal component analysis revealed no differences between the two groups. **(B)** A volcano plot of NMR data revealed no differences between the groups. The dashed red line indicates a *p*-value of 0.05. **(C)** Blood testosterone was not affected by short-term PL treatment.

PLs are key players in many metabolic processes. Thus, we tested whether high-dose dietary PL intake results in a wide range of changes in brain metabolism. In our previous study, both long- and short-term PL supplementations did not affect systemic metabolism, as revealed by blood NMR metabolomic profiling (Saydakova et al., 2023). In this study, we used the same approach to assess brain intermediary metabolism and major neurotransmitter biosynthesis upon the addition of PLs. NMR analysis identified 57 small metabolites in the brain samples, which were further used for PCA and statistical comparison between groups. Neither of the analyses revealed differences between the groups, which is in line with our previous findings (Figures 2A,B).

As testosterone is a key hormone regulating courtship and aggression, and some PLs have been shown to interfere with its biosynthesis, we also measured circulating free testosterone in the blood of the control and test mice (Wang et al., 2023). This experiment revealed that high-dose dietary PL intake did not affect baseline free testosterone levels in test mice (Figure 2C). We only tested testosterone in this study because it is best known to control odor preference and sexual behavior in male mice.

### 3.4 Intranasal PL treatment affects male and female odor discrimination and reduces female preference

**Figure 3:**
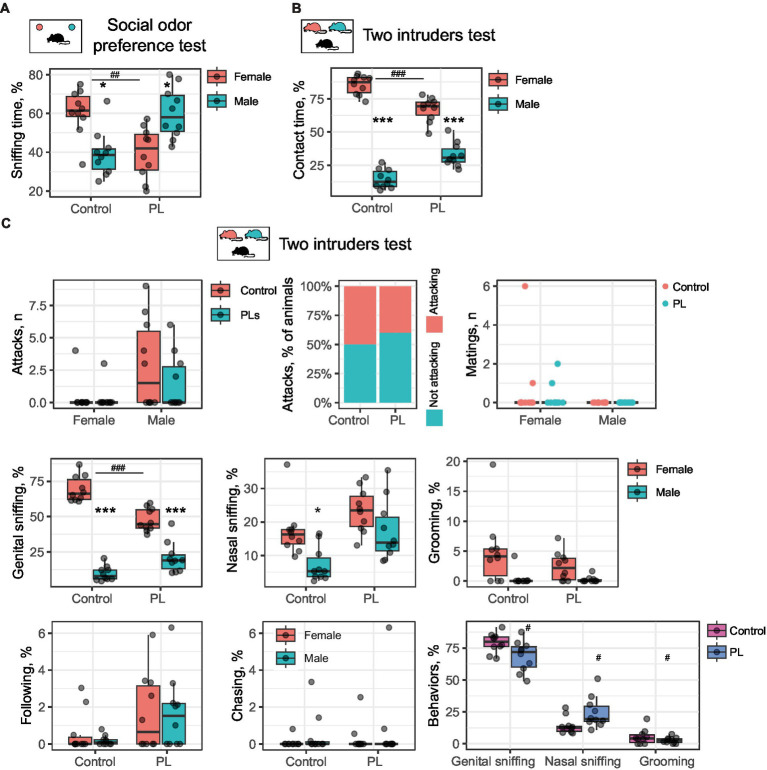
Intranasal PL treatment affects social odor discrimination and reduces female preference. **(A)** Odor preference test. Control mice prefer female bedding, whereas males, after intranasal PL treatment, prefer male bedding samples. ^*^Male vs. female, #control vs. PL. **(B)** The two-intruders test. Intranasal PL treatment decreases the time of contact with a female intruder. ^*^Male vs. female, ^#^control vs. PL. **(C)** Behavioral breakdown of the two-intruders test. ^*^Male vs. female, ^#^control vs. PL. ^*^p < 0.05, ^***,###^*p* < 0.001.

Since PL only impaired interest in social odors but did not affect the perception of other scents, we proposed that they might act within the olfactory system. To examine this hypothesis, we performed intranasal application of the PL mixture 30 min before the test so that PL could not provoke changes in protein expression. Then, we performed the social odor preference test and discovered that a single intranasal application of PL results in a similar behavioral outcome. PL-treated males preferred sniffing the male odor samples rather than the female odor samples [*t*(9) = 2.48, *p*(adjusted) = 0.03]; in contrast, control animals retained interest in female bedding [*t*(9) = 2.88, *p*(adjusted) = 0.03, using Student’s *t*-test for dependent samples]. Intranasal PL treatment also reduced interest in females compared to control animals [*t*(18) = 3.77, *p*(adjusted) = 0.004, using Student’s *t*-test for independent samples] (Figure 3A). Similarly, intranasal PL application also affected resident behavior in the two-intruders test: PL-treated males spent less time investigating females than wild-type males [*t*(18) = 5.07, *p*(adjusted) < 0.001, using Student’s *t*-test for independent samples]. However, they still preferred female over male intruders as well as the control males [control: *t*(9) = 15.78, *p*(adjusted) < 0.001; PLs: *t*(9) = 6.06, *p*(adjusted) < 0.001, using Student’s *t*-test for dependent samples] (Figure 3B).

Unlike the short-term treatment setting, intranasal application of PL did not affect aggression or influence mating behaviors. This result indicates that increased aggression is not an immediate result of PL supplementation. It also suggests that exaggerated aggression is unlikely to reduce interest in females upon PL treatment.

We then questioned which type of social communication had a major impact on intermate contact in the two-intruders test following the intranasal PL application. A behavioral breakdown demonstrates five primary behaviors in addition to attacking and mating. These are genital and nasal sniffing, grooming, following, and chasing (Figure 2C). Genital sniffing is the predominant social activity in male mice within the two-intruders paradigm, and together with nasal sniffing and grooming, it comprises approximately 100% of total contact time. Intranasal PL resulted in reduced genital sniffing of female mice as compared to control mice [*t*(18) = 5.69, *p*(adjusted) < 0.001, using Student’s *t*-test for independent samples], while males from both groups generally preferred sniffing females [Control: *t*(9) = 15.09, *p*(adjusted) < 0.001; PLs: *t*(9) = 5.77, *p*(adjusted) < 0.001] (Figure 3C). Control males also prefer females for nasal sniffing [*t*(9) = 3.19, *p*(adjusted) = 0.03, using Student’s *t*-test for dependent samples], which was not observed for PL-treated males. However, no between-group difference was observed. Interestingly, when comparing the three major behaviors between groups, we found that PL treatment reduced genital sniffing and grooming and extended nasal sniffing [genital sniffing: *t*(18) = 2.34, *p*(adjusted) = 0.046, nasal sniffing: *t*(18) = 2.33, *p*(adjusted) = 0.046, and grooming: *t*(18) = 1.96, *p*(adjusted) < 0.05, using Student’s *t*-test for independent samples] (Figure 3C). This observation might indicate a shift in odor preference from genital to nasal odors resulting from PL supplementation.

Altogether, these data demonstrate that PL might be capable of interfering with pheromone perception via the olfactory system.

## 4 Discussion

The PLs used in this study (PC, PS, and PA) are considered beneficial for human health and are safely marketed as over-the-counter products. For example, PC intake has been shown to restore neuronal plasticity, contribute to acetylcholine biosynthesis, and improve cognitive performance, as evidenced by patient outcomes and laboratory model studies (Derbyshire and Obeid, 2020; Magaquian et al., 2021; Roy et al., 2022). Similarly, PS has been recognized for its positive impact on brain physiology, which is supported by results from both animal models and human trials, making supplements with PS widely accepted for daily consumption (Kim et al., 2014; Glade and Smith, 2015; Ma et al., 2022). Additionally, PA plays a role as a building block in the synthesis of PLs and is popular among athletes for its ability to enhance strength and muscular endurance by acting as an agonist for the mammalian target of the rapamycin (mTOR) signaling pathway (Foster, 2013; Escalante et al., 2016; Hachem et al., 2023).

There are reports in humans and in mice that PL and their derivative fatty acids might affect olfaction. For instance, Khoury et al. (2021) reported that the nasal arachidonic acid level might be positively associated with olfactory deficiency in humans. Meanwhile, dietary polyunsaturated fatty acids might also affect olfactory function in mice (Khoury et al., 2023). In our previous study in mice, we tested the effect of high doses of PL on intestinal morphology and systemic metabolism. It was revealed that excess PL affects mitochondrial ultrastructure and function in the intestinal epithelium but does not change blood intermediary metabolite concentrations (Saydakova et al., 2023). At the same time, high-dose PL supplemented to the diet prenatally and further over the life course of mice strongly suppressed social odor discrimination and affected social behavior (Boldyreva et al., 2024). The results presented here demonstrate that even a short-term (2-week long) high-dose PL supplementation strongly reduces social odor discrimination and affects female preference in male mice.

Interestingly, PL only affected social odor preference but did not impact non-social odor processing. In mice, smell is the primary sensory modality, and odor cues are recognized by two physically separate sensory epithelial structures: the main olfactory epithelium (MOE) and the vomeronasal organ (VNO) (Lankford et al., 2020). MOE detects volatile odorants and utilizes cyclic adenosine monophosphate (cAMP) as a second messenger, whereas VNO primarily senses pheromones and relies on inositol-1,4,5-trisphosphate (IP3) and diacylglycerol (DAG) biosynthesis via phospholipase C (PLC) (Runnenburger et al., 2002; Trinh and Storm, 2004). VNO is thus key to social behavior, aggression, and mating. Thus, one of the potential mechanisms implies that PL inhibits social odor perception by interfering with IP3 and DAG biosynthesis in VNO by competing for PLC given that dietary PL can easily distribute to tissues and organs. Alternatively, pheromones activate a transient receptor potential canonical (TRPC), which is particularly sensitive to the lipid content. The latter affects TRPC membrane localization and cooperation with PLC (Beech, 2012). Excess circulating PL might inhibit TRPC2 function, which is the major pheromone receptor in rodents (Stowers et al., 2002). Considering that PL supplementation into the olfactory system affected social odor discrimination within 30 min, PL might directly act within MOE or VNO.

Given that PL are at least partially metabolized in the intestine and liver, their derivative fatty acids may be responsible for the observed effect on olfactory function and have similar action on signal transduction as the intact PL. Alternatively, PL could act via distinct mechanisms when applied intranasally or supplemented with food via mechanisms that are yet unknown. Further studies on the molecular mechanisms behind PL interference with membrane receptors are needed to understand the exact cellular events in the olfactory system that occur upon PL treatment.

## 5 Conclusion

One of the limitations of this study is that we used the highest doses of PL that have been tested as safe in rats and humans (EFSA Panel on Food Additives and Nutrient Sources added to Food et al., 2017; EFSA Panel on Food Additives and Flavourings et al., 2020). Thus, it is quite unlikely that many people would ingest such high amount of PLs. At the same time, PLs are available over-the-counter and can be misused by certain groups of people. For instance, bodybuilders and athletes can consume PA food supplements at a dose of approximately 7.5 g per day despite the established safe dosage of approximately 750 mg daily (Hoffman et al., 2012). Another limitation is posed by the particularity of the murine olfactory system, which is the primary sensory system in mice.

Moreover, functional VNO is not common in *Homo sapiens*. Thus, the results described here cannot be directly applied to humans. However, the findings presented here might be important to understanding the physiological relevance of PL as modulators of sensory organs and other systems. In this study, we provided *in vivo* data that could inspire studies on the molecular interactions of PL with transmembrane receptors and second messenger signal transducers and re-evaluate their role in ligand sensing.

## Data availability statement

The datasets presented in this study can be found in online repositories. The names of the repository/repositories and accession number(s) can be found at: Raw NMR spectra, description of specimens and samples and metabolite concentrations are available at the Animal Metabolite Database repository, Experiment ID 239 (https://amdb.online/amdb/experiments/239/, accessed January 11, 2024).

## Ethics statement

The animal study was approved by the Ethics Committee of Scientific Research Institute of Neurosciences and Medicine (protocol #3 from 19.05.2022). The study was conducted in accordance with the local legislation and institutional requirements.

## Author contributions

MM: Writing – review & editing, Writing – original draft. JA: Writing – review & editing, Investigation. OS: Writing – review & editing. LB: Writing – review & editing. YT: Writing – review & editing. EK: Writing – review & editing, Writing – original draft.

## Footnotes

^1^http://www.hmdb.ca

^2^https://www.metaboanalyst.ca/
